# The Association between IGF-1 Polymorphisms, IGF-1 Serum Levels, and Cognitive Functions in Healthy Adults: The Amsterdam Growth and Health Longitudinal Study

**DOI:** 10.1155/2014/181327

**Published:** 2014-07-10

**Authors:** Carmilla M. M. Licht, Lise C. van Turenhout, Jan Berend Deijen, Lando L. J. Koppes, Willem van Mechelen, Jos W. R. Twisk, Madeleine L. Drent

**Affiliations:** ^1^Department of Clinical Neuropsychology, VU University, Van der Boechorststraat 1, 1081 BT Amsterdam, The Netherlands; ^2^Neuroscience Campus Amsterdam, VU University, De Boelelaan 1085, 1081 HV Amsterdam, The Netherlands; ^3^Department of Epidemiology and Biostatistics, The EMGO^+^ Institute for Health and Care Research, VU University Medical Center, Van der Boechorststraat 7, 1081 BT Amsterdam, The Netherlands; ^4^Department of Internal Medicine, Endocrine Section, VU University Medical Center, De Boelelaan 1117, 1081 HV Amsterdam, The Netherlands; ^5^Work and Employment Division, Netherlands Organization for Applied Scientific Research (TNO), Polarisavenue 151, 2132 JJ Hoofddorp, The Netherlands; ^6^Department of Public and Occupational Health, EMGO^+^ Institute for Health and Care Research, VU University Medical Center, Van der Boechorststraat 7, 1081 BT Amsterdam, The Netherlands; ^7^Body@Work, Research Center on Physical Activity, Work and Health, TNO-VU University Medical Center, Van der Boechorststraat 7, 1081 BT Amsterdam, The Netherlands; ^8^Department of Health Sciences, Faculty of Earth and Life Sciences, VU University, De Boelelaan 1085, 1081 HV Amsterdam, The Netherlands

## Abstract

Several studies have demonstrated an association between polymorphisms in the insulin-like growth factor-1 (IGF-1) gene and IGF-1 serum levels. IGF-1 levels have been associated with cognitive functioning in older persons and growth hormone deficient patients. The present study investigates whether IGF-1 polymorphisms, IGF-1 levels, and cognition are interconnected in healthy adults. Data of 277 participants (mean age: 42.4 years) of the Amsterdam Growth and Health Longitudinal Study on IGF-1 promoter polymorphisms, IGF-1 serum level, spatial working memory (SWM), paired associate learning (PAL), and IQ tests were analyzed. (M)ANOVAs were applied to confirm the associations between IGF-1 polymorphisms and IGF-1 levels and between IGF-1 levels and cognition. Three groups were distinguished based on specific IGF-1 polymorphism alleles: a homozygote 192 bp/192 bp genotype, a heterozygote 192 bp/x genotype, and a noncarrier x/x genotype. Although different IGF-1 levels were found for the three genotypes, performance on all cognitive tasks and IQ measures was similar. Despite the associations between IGF-1 polymorphisms and IGF-1 levels, no association was found between cognition and IGF-1 levels. It seems that IGF-1 does not play a role in the cognitive performance of healthy middle-aged adults. Possible, IGF-1 fulfills a more developmental and protective role in cognition which becomes apparent during childhood, old-age, or disease.

## 1. Introduction

Insulin-like growth factor-1 (IGF-1) is an important regulator of cell proliferation, differentiation, and apoptosis, has acute insulin-like metabolic effects, and is important for growth and development throughout the body. The level of IGF-1 peaks during puberty and after which it declines with age. Although the IGF-1 serum level is influenced by many factors, such as nutritional status, liver function, and serum levels of sex steroids and insulin, the secretion of this peptide is mainly regulated by growth hormone (GH) [1]. It has been estimated that up to 60% of the variance in IGF-1 serum level has a genetic basis [2, 3]. Several polymorphisms in the promoter region of the IGF-1-gene have been identified, comprising a variable length cytosine-adenine (CA) repeat sequence [4, 5]. These polymorphisms are thought to influence the transcription rate of IGF-1, which in turn affects serum IGF-1 levels [6–8]. The 192 bp allele is the most common allele and therefore is called the wild type [6, 7, 9, 10]. Results of studies that evaluated the relationship between the IGF-1 promoter polymorphisms and IGF-1 levels are contradictory; the homozygote 192 bp genotype has been associated with both higher and lower IGF-1 levels compared to the heterozygote and noncarrier genotypes [6–8, 11, 12].

A review by Fernandez et al., 2007, emphasizes the importance of IGF-1 for the whole body and especially for the brain. The authors summarize IGF-1's actions, which appear to be not only on a molecular level, but also on a more general level, with effects on nutrient supply, amyloid clearance, resilience to insult, behavior, and cognitive status. Altogether, IGF-1 seems to be essential for normal brain function and cognition [13], although the exact mechanisms remain unclear. Studies investigating GH substitution therapy in GH-deficient patients, leading to an increase in serum IGF-1, have indeed reported that long-term memory and working memory improved in these patients [14–17]. As cognitive functions as well as IGF-1 level decline with age and as several signs of brain aging, such as the accumulation of deleterious compounds and reduced neurogenesis and angiogenesis, are analogous to effects of IGF-1 deficiency, IGF-1 might be an important factor connecting the aging of the brain to impaired cognitive functioning. A meta-analysis on the association between IGF-1 and cognition in the healthy elderly indicated that IGF-1 is positively associated with overall cognition [13, 18–21]. The authors of this meta-analysis also indicated that a great variety of cognitive tests were used and the somatotrophic axis may influence some cognitive processes but not all. Since IGF-1 receptor densities differ throughout the brain [22], some cognitive domains may correlate stronger with IGF-1 levels than others. For instance, IGF-1 receptors are more abundant in hippocampal regions than in frontal areas. Performance on cognitive tasks that require much hippocampal input may therefore be more influenced by IGF-1 levels than tasks that require more frontal involvement.

Although associations between IGF-1 and cognitive performance as well as between IGF-1 polymorphisms and IGF-1 serum levels have previously been found, studies were limited to GH-deficient patients and the healthy elderly. The question of whether the IGF-1 polymorphisms and the polymorphism-related differences in IGF-1 serum levels are also associated with cognitive performance in healthy and younger adults remains unanswered to date. Therefore the present study investigated the association between polymorphisms of the IGF-1 promoter gene, IGF-1 levels, and cognitive functioning in a large sample of healthy adults aged 41–47 years. Cognitive tasks were included measuring brain activity in temporal and frontal areas of which is known that IGF-1 receptors are present. Additionally, IQ measures were included to assess more general cognition.

## 2. Material and Methods

### 2.1. Study Population and Study Design

Data of the ongoing Amsterdam Growth and Health Longitudinal Study (AGAHLS) were used. This study started in 1976 with 698 subjects at the age of approximately 13 years and was designed to increase the knowledge of growth, development, and health of children in adolescence. In 2000, 375 subjects still participated in the AGAHLS. In these subjects, the genetic polymorphisms of the IGF-1 gene were determined. As the distribution of the IGF-1 polymorphisms in non-Caucasian subjects is different from that in Caucasian subjects [6, 23] and ethnic differences in IGF-1 levels have been reported [23, 24], 31 non-Caucasian subjects were excluded. Of the 344 subjects with data of genetic polymorphisms, IGF-1 levels were determined in serum samples drawn in 2006 in the 287 subjects that still participated at that time. Cognitive functioning and IQ were also measured in 2006; however 8 subjects had missing data on these variables. Finally, for 1 subject health data were not collected. The remaining sample of 278 subjects did not suffer from diseases that might interact with IGF-I serum levels; for example, no subjects had a history of diabetes mellitus and renal or liver failure. One subject used medication for thyroid disorder. Moreover, subjects did not report a history of alcohol abuse. A number of 53 females used oral contraceptives. A number of 41 subjects reported cigarette smoking in 2000 (no complete data is available for smoking in 2006).

In sum, for the present study, 278 Caucasian subjects (131 men, 147 women) complete data on the IGF-1 promoter polymorphism genotype, IGF-1 serum level, scores on the cognitive function tests, and information on health, lifestyle, and psychological factors were available for analysis.

### 2.2. Genotyping

The genetic polymorphisms of the IGF-1 gene were determined as previously described [7]. In brief, DNA was isolated using standard methods. Polymerase chain reaction (PCR) was performed in a final volume of 10 *μ*L containing 10 ng DNA, 10∗ Gold (Au) buffer (Perkins and Elmer), 200 M dNTP, 30 pmol of each primer, 3 mM MgCl_2_, and 0.5 U Ampli Tag Gold polymerase (Perkins and Elmer). The PCR program consisted of 5 min of denaturation at 95°C followed by 30 cycli of 30 sec 95°C, 30 sec 55°C, and 30 sec 72°C with an extension of 10 min at 72°C after the last cycle. Forward primers were labeled with FAM [4] to determine the size of the PCR products by fragment analysis (ABI-Prism genetic analyzer with Genescan 2.1 software). The Genescan 350/500 Tamra was used as internal size standard within the fragment analysis [10].

### 2.3. IGF-1 Serum Levels

IGF-1 serum levels were measured using a commercially available assay (Chemiluminescent immunometric, Immulite 2500, DPC, Los Angeles, USA). Samples were stored at −80°C. All samples were analyzed in one central laboratory using the same IGF-I assay. As the subjects were healthy, we did not expect abnormalities in the binding proteins. The IGF-I assay was calibrated with reference to the 1st WHO international standard 02/254. The detection limit was 3.2 nmol/L. The intra-assay coefficient of variation was 5% for the entire range. The interassay coefficient of variation was 5% for the entire range.

### 2.4. Assessments of Cognitive Function and IQ

Spatial working memory (SWM) and paired associate learning (PAL) were assessed using standard procedures from the Cambridge Neuropsychological Automated Testing Battery (CANTAB) [25]. The spatial working memory (SWM) test, sensitive for frontal lobe dysfunction, is a self-ordered search task in which subjects must find a blue square which is hidden in a box on the screen. A number of boxes are presented on the screen and the subject must touch each box in turn to discover where it is hidden (a search). Subjects received four test trials with each of 2, 3, 4, 6, and 8 boxes. Subjects are informed that once a counter is found, that box will not hide another one in that search set. In the present study we evaluated “between errors” and “within errors” for 8-box problems, the most difficult and most sensitive part of the task. “Between errors” are defined as the number of times the subject revisits a box in which a token has previously been found. “Within errors” are defined as the number of times a subject revisits a box already found to be empty during the same search. Individual differences in the ability to adopt a consistent search sequence on the more difficult 6- and 8-box trials were also evaluated (strategy score) [26]. Higher scores on SWM subtests reflect a worse performance.

The paired associate learning (PAL, visual memory) test is sensitive for medial temporal lobe functioning: six boxes appear and each opens in sequence showing up to 8 patterns. Subjects are then shown a pattern in the middle of the screen and asked to choose the box which the pattern previously appeared in [27]. We evaluated the following PAL parameters, in which higher scores reflect a worse performance: total number of errors, total number of errors on the 8-pattern trials, and total number of trials.

The Groninger intelligence test (GIT) is the commonly used Dutch estimate of formal IQ [28]. A short version was used, consisting of the subtests “Legkaart” (spatial jigsaw puzzles), “Cijferen” (arithmetic), and “Woordmatrijzen” (word matrices). In addition, the “Nederlandse Leestest voor Volwassenen” (NLV), a Dutch version of the National Adult Reading Test (NART), was carried out [29]. The NLV is a measure of verbal intelligence [30].

### 2.5. Covariates

As covariates we included age in years (at the 2006 assessment), gender, and body mass index (BMI) defined as the weight in kilograms divided by the square of the height in meters.

### 2.6. Data Analyses

The general characteristics of the sample were analyzed using chi-square and ANOVA statistics. Mean cognitive scores per genotype were analyzed with ANOVAs for the IQ measures and with MANOVAs for the different parameters of the SWM and PAL and adjusted for age, gender, and BMI.

Correlations between IGF-1 levels and cognitive scores were obtained with Pearson's correlation (2-tailed). To investigate nonlinear associations between IGF-1 levels and cognitive scores, subjects were assigned to five groups based on their IGF-1 level. Quintiles were made for men and women separately based on similar group size and merged afterwards. Mean cognitive scores per quintile were compared using MANOVA (PAL and SWM) and ANOVA (GIT and NLV) adjusted for age, gender, and BMI.

Statistical analyses were carried out using SPSS version 20.0 (IBM, Chicago).

## 3. Results

First, all IGF-1 promoter alleles were analyzed. The allele frequency of the 192 bp allele was 64.0% (356 alleles), corresponding to the allele frequency reported in other Caucasian populations, estimated to be between 59% and 70% [6, 7, 10, 11, 31]. In line with former reports [7, 10], the allele frequency we found for the 194 bp allele was 19.6% (109 alleles). Frequencies of occurrence of the remaining alleles were as follows: 36 alleles of 196 bp, 28 alleles of 190 bp, 16 alleles of 189 bp, 9 alleles of 198 bp, and 2 alleles of 176 bp. We distinguished 17 different genotypes: 176/192 (*n* = 1), 176/196 (*n* = 1), 188/192 (*n* = 9), 188/194 (*n* = 4), 188/196 (*n* = 3), 190/190 (*n* = 1), 190/192 (*n* = 16), 190/194 (*n* = 5), 190/196 (*n* = 3), 190/198 (*n* = 2), 192/192 (*n* = 109), 192/194 (*n* = 84), 192/196 (*n* = 23), 192/198 (*n* = 6), 194/194 (*n* = 3), 194/196 (*n* = 7), and 194/198 (*n* = 1), as was done by van Turenhout et al. 2011 [8]. Since the 192 bp allele was the most frequent one, genotypes were further divided based on the presence of the 192 bp allele and in this way three groups were distinguished as was done before [31–34]: a homozygote 192 bp group (*n* = 109, 39.2%), a heterozygote 192 bp group (*n* = 138, 49.6%), and a group carrying alleles other than 192 bp (*n* = 31, 11.2%). The distribution of genotypes was in Hardy-Weinberg equilibrium (*χ* ^2^ = 1.68, df = 1, *P* = 0.19).

Table 1 shows the main sample characteristics per genotype. Age, gender, and BMI did not differ between genotypes. Unadjusted ANOVAs showed that IGF-1 levels differ between the homozygote, the heterozygote, and the noncarrier groups. The homozygote 192/192 genotype had the lowest IGF-1 level (22.6, SD = 5.4). The heterozygote 192/x group and the noncarrier group both had a significant higher mean IGF-1 level compared to the homozygote group (24.4, SD = 5.5, and 25.1, SD = 5.4, respectively, overall *P* = 0.01).

The mean scores on all cognitive measures per genotype are represented in Table 2. This table shows that, unadjusted or adjusted for age, gender, and BMI, no significant differences in cognitive functioning are present between genotypes. In addition, neither consistent trends nor interactions with gender were found.

**Figure 1:**
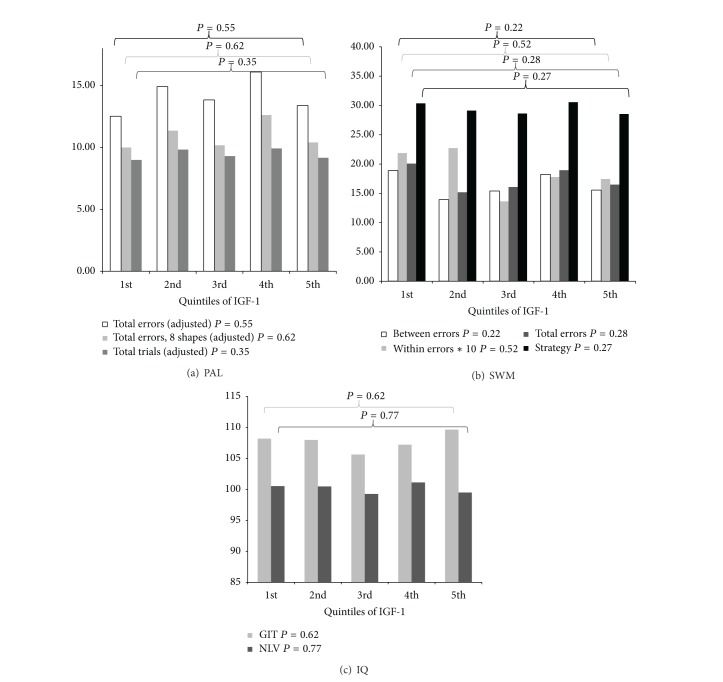
Mean adjusted∗ cognitive scores per quintile of IGF-1 serum level. Quintiles of IGF-1 were made for men and women separately and merged afterwards. *P* values represent overall *P* values based on ANOVA statistics for the comparison of the GIT and NLV between quintiles of IGF-1 and MANOVA statistics for the comparison of PAL and SWM scores between quintiles of IGF-1. ∗Adjusted for age, gender, and BMI.

Correlations between IGF-1 level and cognitive scores are described in Table 3. No significant correlations were found. In addition, the directions of the correlations were opposite between tasks. To investigate a more nonlinear association, cognitive and IQ scores were compared between quintiles of IGF-1 levels. The results of ANOVAs and MANOVAs adjusted for age, gender, and BMI are presented in Figures 1(a)–1(c). All  *P* values for overall comparison were ≥0.22 and in-depth comparison of all quintiles also revealed no significant differences. Results and figures were similar for men and women.

## 4. Discussion

This study explores the cross-sectional relationship of the IGF-1 promoter polymorphisms and IGF-1 serum level with cognition. We found that subjects with the homozygote 192 bp genotype had the lowest IGF-1 serum levels, followed by the heterozygote genotype and the noncarrier genotype. The result that the presence of the 192 bp allele is associated with lower IGF-1 levels is in line with most previous findings reviewed by Fletcher et al. [6], although it is in contrast with some other studies that report opposite associations [7, 11, 32]. Two studies have indicated that the decrease in IGF-1 levels normally seen with age differs between these three genotypes [11, 35]. Therefore, the age at which the association between IGF-polymorphisms and IGF-1 level is investigated might partly determine the outcome. For instance, if the IGF-1 level of homozygote subjects decreases the most over time compared to other genotypes, our homozygote adults (with low IGF-1 levels) could have had relatively high levels at young age, which would have revealed a different association.

The IGF-1 promoter polymorphisms were not associated with cognition. As far as we know, we are the first to study this association. Although we expected that IGF-1 promoter polymorphisms, which regulate IGF-1 transcription, are related to cognition* via* IGF-1 serum levels, this appears not to be the case in our middle-aged adult sample.

In addition, IGF-1 serum levels were also not associated with cognition. These findings are in contrast with several previous studies that did find an association between IGF-1 levels and cognitive functions. The meta-analysis of Arwert et al. [18] and several more recent studies [36–38] report a (overall) positive correlation between IGF-1 levels and cognitive performance in healthy older people. In addition, associations between IGF-1 and cognition also have been found in growth hormone deficient children [39] and adults [15, 19], children with infantile spasms [40], people with Parkinson's disease [41], Alzheimer [42], and delirium [43]. An important difference between these studies and ours is that we investigate the relationship between IGF-1 levels and cognition in healthy adults, whereas all previous studies used elderly or patient groups. The apparent contradiction in findings might therefore suggest that IGF-1 levels do not play a role in (relatively good) cognitive performance in a healthy adult group within the same age range. Moreover, our healthy sample had IGF-1 levels within a normal range and differences in cognitive functions might not be reflected by minor variations in IGF-1 levels but only at substantially higher or lower IGF-1 levels, seen in, for example, GH-deficient and acromegalic patients. However, findings of a study on neurocognitive impairments in acromegalic patients indicated that cured acromegaly patients still displayed specific memory deficits. Patients included in the study had been cured of acromegaly at a minimum of the preceding 6 months before the neurocognitive assessments. Thus, there was no evidence of full recovery from neurocognitive impairment after cure of acromegaly in patients with short-term biochemical remission [44]. It may well be true that the preserved neurocognitive deficits after postoperative biochemical remission are due to prolonged GH/IGF-I hypersecretion before surgery. As the underlying mechanisms of the neurocognitive deficits are not known yet, it may be suggested that in spite of the normalised secretion of GH/IGF-I some kind of neurological damage is still present.

Another interesting hypothesis postulated that IGF-1 might have different roles in the human body: a* developmental *role that focuses, for instance, on longevity, somatic growth, and insulin signaling and a* protective *role that regulates, for example, body composition, blood flow to the brain, and neuronal survival. The hypothesis suggested that working mechanisms and goals of developmental and protective effects of IGF-1 are entirely different or even contradictory and alternate in dominating at different ages [45]. If this hypothesis is confirmed, a possible explanation for our findings is that IGF-1 influences cognition only by its involvement in the development of the brain from fetal stage up to puberty and by its neuroprotective effects in the elderly but does not correlate with cognition in adulthood. Although it seems justifiable to conclude that interventions set at increasing IGF-1 levels for a better cognitive performance do not seem useful for these healthy adults, IGF-1 levels during adulthood may still contribute to cognitive performance at old-age. Regarding the previously mentioned evidence that different IGF-1 polymorphisms show differential decreases in IGF-1 level with aging [11, 35], IGF-1 levels might not correlate with cognition in adulthood but might do in old-age.

This study has several strengths. The different cognitive tasks that were included enabled us to investigate frontal, temporal, and more general cognition. Since IGF-1 receptor densities differ throughout the brain [22], we were able to study whether cognitive performance on tasks that mainly involve a brain area with high IGF-1 receptor densities (the temporal lobe) is different from the performance on a task involving a brain area with lower IGF-1 receptors densities: the frontal cortex. However, no differences were detected suggesting that the amount of IGF-1 receptors in a brain region does not play a role in the association between IGF-1 level and cognitive performance in healthy adults.

Furthermore, an approximately equal amount of men and women participated, so confounding and interaction effects with respect to sex could be investigated. Finally, IGF-1 levels and IGF-1 polymorphisms, as well as cognitive measures, were collected so the possible mediating role of IGF-1 levels in the association between IGF-1 polymorphisms and cognition could be investigated. Some limitations have to be acknowledged as well. The study sample has a small age range, which prevented us from studying this association in the broader adult age range. That means that the present results can only be generalized to a population of males and females around the age of 42. In addition, sample size was too narrow to study all separate—sometimes rare—genotypes of the IGF-1 polymorphism. However, since IGF-1 levels did not associate with cognition, a different arrangement of genotype groups would not have changed our findings.

In conclusion, our population-based study on Caucasian individuals with an average age of 42 confirms the association between IGF-1 polymorphisms and IGF-1 levels but found no association between IGF-1 polymorphisms and IGF-1 levels with cognitive functioning. Our results indicate that, in a sample of healthy middle-aged adults, IGF-1 serum levels are not associated with cognitive performance. These findings suggest that the serum IGF-1 levels work as a cognitive developmental factor during childhood and as a protective factor for cognition in older persons or patients rather than associate with cognition in healthy adults.

## Figures and Tables

**Table 1:** Sample characteristics.

	Homozygotic 192/192 *N* = 109	Heterozygotic 192/x *N* = 138	Noncarrier x/x *N* = 31	P*
Sociodemographics				
Age, mean years (SD)	42.4 (0.8)	42.3 (0.7)	42.4 (0.9)	0.76
% female	56.0	50.7	51.6	0.71
BMI, mean kilograms/meters^2^ (SD)	24.5 (3.7)	24.8 (3.8)	24.6 (2.7)	0.78
Alleles				
176, *N*	0	1	1	—
186, *N*	0	0	0	—
188, *N*	0	9	7	—
190, *N*	0	16	12	—
192, *N*	218	138	0	—
194, *N*	0	84	25	—
196, *N*	0	22	14	—
198, *N*	0	6	3	—
IGF-1, mean nml/liter (SD)	22.6 (5.4)	24.4 (5.5)	25.1 (5.4)	0.01

*Based on *χ* ^2^ and ANOVA statistics for dichotomous or categorical and continuous measures, respectively.

SD: standard deviation, BMI: body mass index, PAL: paired associates learning, SWM: spatial working memory, GIT: Groninger intelligentietest test, and NLV: Nederlandse Leestest voor Volwassenen.

**Table 2:** Mean unadjusted and adjusted∗ scores on cognitive measures per genotype of IGF-1 promoter region (*N* = 278).

	Homozygotic 192/192	Heterozygotic 192/x	Noncarrier x/x	*P*
	*n* = 109	*n* = 138	*n* = 31	
*Unadjusted *				
PAL				
Total error adjusted, mean (SD)	12.80 (8.7)	15.40 (13.5)	13.71 (11.9)	0.22^a^
Total error 8-box adjusted, mean (SD)	10.01 (7.5)	12.01 (11.8)	9.58 (8.4)	0.21^a^
Total trials, mean (SD)	9.14 (2.5)	9.75 (3.1)	9.35 (3.2)	0.25^a^
SWM				
Between errors, mean (SD)	17.28 (14.6)	15.14 (11.9)	18.52 (16.5)	0.30^a^
Within errors mean (SD)	1.94 (3.4)	1.81 (2.8)	1.90 (2.2)	0.94^a^
Total errors, mean (SD)	18.23 (15.3)	16.15 (12.3)	19.52 (16.9)	0.34^a^
Strategy, mean (SD)	29.79 (6.2)	29.22 (6.4)	29.19 (7.4)	0.77^a^
IQ				
GIT, mean (SD)	108.32 (13.4)	106.86 (13.0)	109.00 (13.3)	0.58^b^
NLV, mean (SD)	99.61 (8.1)	100.19 (9.3)	102.23 (8.0)	0.34^b^
*Adjusted* ∗				
PAL				
Total error adjusted, mean (SE)	12.86 (1.1)	15.36 (1.0)	13.50 (2.1)	0.24^a^
Total error 8-box adjusted, mean (SE)	10.06 (1.0)	11.98 (0.9)	9.39 (1.8)	0.22^a^
Total trials, mean (SE)	9.15 (0.3)	9.74 (0.2)	9.26 (0.5)	0.27^a^
SWM				
Between errors, mean (SE)	17.04 (1.2)	15.28 (1.1)	18.53 (2.4)	0.36^a^
Within errors mean (SE)	1.92 (0.3)	1.83 (0.3)	1.97 (0.5)	0.96^a^
Total errors, mean (SE)	17.98 (1.3)	16.31 (1.2)	19.57 (2.5)	0.40^a^
Strategy, mean (SE)	29.67 (0.6)	29.29 (0.5)	29.28 (1.1)	0.88^a^
IQ				
GIT, mean (SE)	108.34 (1.2)	106.89 (1.1)	109.50 (2.4)	0.51^b^
NLV, mean (SE)	99.43 (0.8)	100.31 (0.7)	102.40 (1.5)	0.23^b^

PAL: paired associates learning, SWM: spatial working memory, GIT: Groninger intelligentietest test (Groninger intelligence test), and NLV: Nederlandse Leestest voor Volwassenen (Dutch reading test for adults).

^a^Based on multivariate analysis of variance.

^b^Based on univariate analysis of variance.

∗Adjusted for age, gender, and BMI.

**Table 3:** Correlations between cognitive measures and IGF-1 levels (*n* = 278).

	IGF1 2006 in nmol/L	
	Correlation coefficient	P*
PAL		
Total errors (adjusted)	0.031	0.61
Total errors (8 shapes, adjusted)	0.032	0.60
Total trials (adjusted)	0.014	0.81
SWM		
Between errors	−0.029	0.63
Strategy	−0.044	0.46
Total errors	−0.037	0.54
Within errors	−0.053	0.38
IQ		
GIT	0.051	0.40
NLV	−0.014	0.82

*2-tailed.
